# An innovative fuzzy model control strategy for Trans-Z-Source DC-DC boost converters in satellite power systems

**DOI:** 10.1038/s41598-025-28748-2

**Published:** 2025-12-30

**Authors:** Mostafa Wageh Lotfy, Aziza I. Hussein, Mohammed M. Abo-Zahhad, M. Mourad Mabrook

**Affiliations:** 1 Department of Process Control Technology, Faculty of Technology and Education, Beni-Suef University, Beni-Suef, Egypt; 2 Electrical & Computer Eng. Dept, Effat University, Jeddah, Saudi Arabia; 3 Department of Electrical Engineering, Faculty of Engineering, Sohag University, Sohag, New Sohag City, Egypt; 4 Space Communication Dept, Faculty of Navigation Science & Space Technology, Beni-Suef University, Beni-Suef, Egypt

**Keywords:** DC/DC converter, DC/DC boost converter, Quasi-Trans-Z-source network, Fuzzy model control (FMC), Satellite systems, Energy science and technology, Engineering

## Abstract

This paper investigates the critical role of Direct Current to Direct Current (DC-DC) converters in satellite power systems, emphasizing the need for efficient and reliable energy conversion under dynamic space conditions. A novel Fuzzy Model Control (FMC) strategy is proposed for a quasi-Trans-Z-Source DC-DC boost converter, addressing the inherent challenges of nonlinear power dynamics and multiport energy flow management in space applications. The proposed approach enhances mode transitions, improves solar energy extraction from photovoltaic (PV) panels, and ensures stable voltage regulation under fluctuating load conditions. A comprehensive theoretical analysis of the circuit topology highlights its advantages over conventional boost converters, including continuous input current, higher voltage gain, and reduced passive component stress. Simulation and experimental results demonstrate that the proposed FMC achieves ± 1.5% voltage regulation accuracy, reduces current ripple by 28%, and improves transient response time by 35% compared to a conventional Proportional-Integral (PI) controller. The overall system efficiency reaches 94.7% under nominal conditions. Furthermore, the control strategy effectively manages constraints such as duty cycle limits and dynamic disturbances, confirming its real-time applicability for spaceborne platforms such as nanosatellites and CubeSats.

## Introduction

DC converters are crucial components in satellite systems, ensuring efficient power management and distribution to support the reliable operation of various subsystems in the harsh space environment^1^. They enable the conversion of electrical power between different components, ensuring compatibility and optimal performance. The DC converter regulates and optimizes the power generated by the solar input panel for use within the satellite system^2,3^. Designed for high efficiency and reliability, DC converters in satellite systems are essential to maximize power utilization and ensure uninterrupted operation in space^4^.

With the rising need for energy conservation and rise in the use of renewable energy systems in the power system, there has been interest in enhancement of power electronics components; this include the control strategies for the DC-DC boost converters^5,6^. A primary purpose of these converters is put to use in renewable energy systems like solar and wind power, electric cars, and portable electronics to boost power conversion and dependability in systems underway. Due to the varying demands of power systems, new methods of controlling power systems are being sought by researchers and engineers, including Artificial Neural Network (ANN) for solving problems related to DC-DC boost converters^7,8^. The main functionality of the power and electrical subsystem is to provide sufficient power load to all the satellite subsystems. Over the centuries, people have always started launching satellites into orbit in response to rising needs around the world. These satellite has some of its uses as a mean of communication, astronomy, military use, weather forecasting, and many others.

Moreover, the sphere of satellite technology has actively developed due to the usage of nanosatellites and CubeSats. These small-scale satellites have proffered a new solution model in space exploration that envisage improved mobility, ease of access and affordable means of satellite deployment and control^9,10^. A nanosatellite can also be known as a nanosat and is generally described as a satellite that has a weight of between 1 and 10 kg. Despite their relatively modest size, the missions’ overall capabilities comprise multiple categories, such as scientific investigation, Earth monitoring and space exploration, communications, and technology experimentation. Nanosatellites in general and CubeSats in particular, are lightweight by design, and are constructed according to a standardized modular structure based on cubic units of 10 cm on each side. These “cubes” can be combined to develop larger satellite arrangements like 1U which is simply 1 unit, 2U, 3U and on, making it possible to design satellites that are optimized for different uses^11^.

Recent advances such as nano satellites and CubeSats have made it easier for many other stakeholders such as educative institutions, research facilities and even commercial companies to engage in space travel missions^16^. Compact sizes and modular structures have transformed the S/C design and launch perspectives and encouraged active advancements and cooperation within the Aerospace field. In addition, the reduced costs of nano satellites and CubeSats makes it easier for interested enthusiasts and new space industry entrants to access space and experiment without significant capital investment^17^. Nano satellites and CubeSats are many in number and are comparatively small in size; however, they are equipped with numerous payloads and instruments, which include cameras and sensors, communications equipment and scientific instruments^18^. Such payloads facilitate various systems such as Earth observation, remote sensing, climate assessment, and disaster relief. In addition to this, CubeSats can easily be adapted for many different uses since their architecture is very modular with thin layers of software over hardware allowing for easy alterations, customizations and experiments^19^. A characteristic feature of nano satellites and CubeSats is their work in constellations, where satellites are collectively focused on the fulfillment of tasks. Multi-satellite missions based on constellation principles have a number of advantages over the implementation of single-satellite missions, such as improved coverage, increased redundancy, and increased information acquisition potential. Thus, using satellite-distributed networks, the researches can deliver overall and real-time information on different phenomena, including the changes in the environment, the atmospheric processes^20^.

**Fig. 1:**
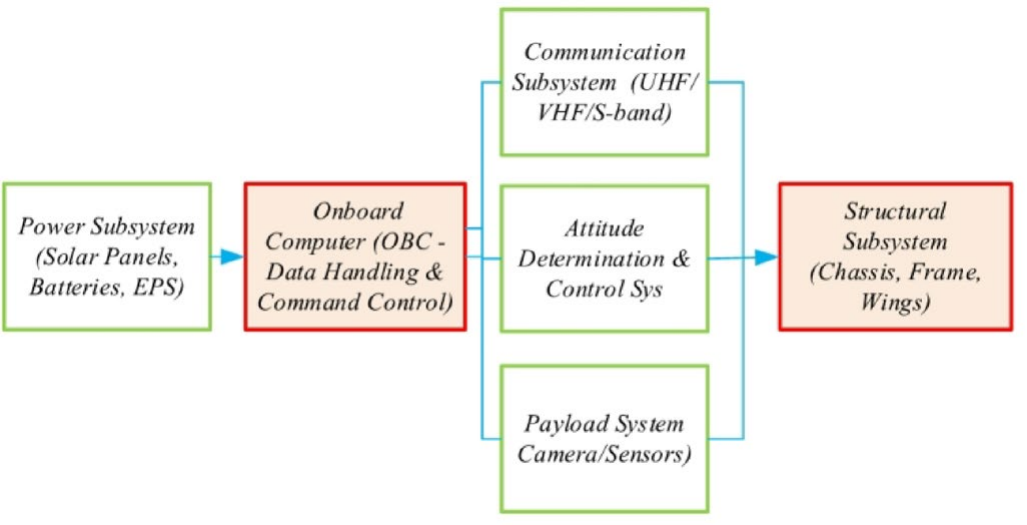
Block diagram of the cubesat system architecture.

In recent years nanosatellites and CubeSats have been used in different missions and projects including scientific, technological and educational. Such as the scientific exploration of Earth magnetosphere, controlling the agricultural cycles, and the experimental analysis of novel forms of propulsion systems through the utilization of cubesats. Further, nanosatellites have been used to improve communication infrastructure especially in the areas that are hardly reached or lacked adequate telecommunication networks, offering internet accessibility and telecommunication facilities to communities all over the world^21–23^. The small satellite currently being used is the CubeSat 3, depicted in Fig. 1 below. This figure illustrates the block diagram of the CubeSat system architecture, highlighting the key subsystems integrated into the platform.

In modern satellite power systems, efficient and reliable energy conversion is crucial to ensure continuous operation under highly dynamic space conditions. This study proposes a Fuzzy Model Control (FMC) strategy for a quasi-Trans-Z-Source DC-DC boost converter to address the challenges of nonlinear power dynamics and multiport energy flow management. The proposed method improves solar photovoltaic (PV) energy extraction, ensures stable voltage regulation under fluctuating loads, and enables smooth mode transitions. Compared to conventional boost converters, the quasi-Trans-Z-Source topology offers continuous input current, higher voltage gain, and reduced stress on passive components. Simulation and experimental validation demonstrate that the FMC achieves ± 1.5% voltage regulation accuracy, reduces current ripple by 28%, and enhances transient response by 35%, reaching an overall efficiency of 94.7%. These results highlight the control strategy’s effectiveness for real-time applications in nanosatellites and CubeSats. The DC-DC converters are vital in power electronics systems where they provide the power conversion ingredients and the control means for converting electrical power. Analyzing the various types of DC-DC converter available, Quasi-Trans-Z-Source network can be recognized as the one of the most innovative due to a number of its advantages in parameters of performance, efficiency and flexibility^12,13^. To introduce the structure of the Quasi-Trans-Z-Source network, it includes the difficulties as well as the specifications of the conventional DC-DC converters. The problem that causes output voltage instabilities for conventional converters includes input voltage variations, low voltage gain, and low efficiency problems. These challenges are dealt with by the development of a new solution; the Qua-Trans-Z-Source network which incorporate the qualities of the Trans-Z-Source networks and other modifications^14^. The Trans-Z-Source network, which serves as the foundation for the Quasi-Trans-Z-Source network, is known for its unique impedance characteristics. Unlike conventional impedance networks, the Trans-Z-Source network offers enhanced voltage-boosting capabilities, making it suitable for applications that require high voltage gain^15^.

**Table 1:** Comparative features of conventional boost, Z-Source/Quasi-Z-source, and proposed Quasi-Trans-Z-Source converter.

Feature/Topology	Conventional Boost Converter^2^	Z-Source/Quasi-Z-Source Converter^7^	Proposed Quasi-Trans-Z-Source with FMC
Voltage Gain	Limited (depends heavily on duty cycle)	Moderate, improved over boost	High, with extended boosting capability
Input Current	Discontinuous at high duty cycle	Continuous	Continuous, reducing PV stress
Component Stress	High voltage stress on switch and diode	Moderate	Reduced, enhancing reliability
Control Strategy	PI-based (slower response)	PI/Fuzzy (limited adaptability)	Fuzzy Model Control (fast, robust, adaptive)
Current Ripple	Relatively high	Reduced compared to boost	28% reduction compared to PI-based control
Transient Response	Slower	Improved	35% faster than PI control
System Efficiency	88–91% (typical)	~ 92%	94.7% under nominal conditions
Suitability for Space Applications	Limited (due to reliability and ripple issues)	Moderate	Highly suitable for nanosatellites and CubeSats

Despite significant progress in DC–DC converter topologies and intelligent control methods, their application to satellite power systems remains limited. Conventional boost and Z-source converters suffer from low voltage gain, discontinuous input currents, and high switching stresses, which make them unsuitable for the stringent requirements of nanosatellites and CubeSats. Moreover, most existing control techniques such as Proportional–Integral (PI) and Sliding Mode Control lack robustness under nonlinear dynamics, fluctuating loads, and radiation-induced variations common in orbital environments. While fuzzy logic–based controllers have been explored in terrestrial power systems, their integration with quasi-Trans-Z-Source (q-TZSC) converters in satellite power management has not been fully investigated, particularly with experimental validation. This gap highlights the need for a novel control strategy that combines intelligent adaptability with improved converter topology to achieve high efficiency, stable voltage regulation, and real-time applicability in space borne platforms.

The Quasi-Trans-Z-Source network retains essential components from the original Y-source network, such as the three-winding coupled inductor (N1, N2, and N3) and capacitor C1. However, it introduces new elements, including a DC-blocking capacitor (C2) and an input inductor (LIn). These changes are designed to address the issues faced by earlier Multi-Cell Impedance Source (MCIS) networks, ensuring smoother operation and higher efficiency^12^.A significant advancement in the Quasi-Trans-Z-Source network is the development of various two-winding quasi-networks. These networks, characterized by continuous input currents, offer advantages in terms of mathematical derivations for parameters and voltage gains. The flexibility of these quasi-networks enhances their adaptability for different applications.

To further clarify the justification of the proposed design over traditional converters, a comparative analysis is presented in Table 1. The conventional boost converter suffers from limited voltage gain, discontinuous input current at high duty cycles, and increased stress on switching devices, which reduce its suitability for demanding satellite applications. Although Z-Source and quasi-Z-Source converters address some of these issues by providing moderate voltage boosting and continuous input current, they still experience limitations in terms of component stress and control flexibility. In contrast, the proposed quasi-Trans-Z-Source DC–DC boost converter, when integrated with the Fuzzy Model Control (FMC) strategy, provides enhanced voltage gain, continuous input current, reduced current ripple, and superior transient response. Furthermore, its high efficiency (94.7% under nominal operating conditions) and reliability make it particularly well-suited for nanosatellite and CubeSat platforms where compactness, robustness, and adaptability to dynamic space environments are critical.

While the design requirements for the quasi-T-Source or Quasi–Trans-Z-Source network align with those of conventional counterparts, the same cannot be said for the quasi-Γ-Z-Source network. The quasi-Γ-Z-Source network introduces new winding design specifications, demonstrating the versatility and adaptability of the Quasi-Trans-Z-Source network to meet specific system demands^13^.The Quasi-Trans-Z-Source network excels in achieving high voltage gain, a crucial factor in improving the efficiency of DC-DC converters. The peak output voltage during the non-shoot-through state is a key parameter, with the network voltage gain derived based on the winding factor. The application of the Quasi-Trans-Z-Source network spans a variety of domains, including renewable energy systems, electric vehicles, and power distribution. Its ability to address the challenges of traditional converters, along with its continuous input current characteristic, makes it a promising solution for modern power electronics applications.

The paper is structured as follows: Section II offers an overview of the satellite power system operation. The study begins with an introduction and then moves to a detailed discussion of the DC-DC converter in Section III, which also covers the Fuzzy Logic controller modulation approach. Section IV presents the results of the simulations conducted using a MATLAB/Simulink model. Finally, the experimental results and conclusions are discussed in the last section.

## Satellite power system operation

In the field of satellite operations, there is a constant drive to enhance onboard processing capabilities to meet the increasing demands of modern technology. Satellite operators are increasingly turning to advanced ultra-deep-submicron Field-Programmable Gate Arrays (FPGAs) and Application-Specific Integrated Circuits (ASICs) to fulfill these needs. However, these sophisticated components come with significant power demands, requiring low voltage and high current. As a result, original equipment manufacturers (OEMs) face the challenge of delivering enhanced functionality within smaller payloads and platforms while ensuring cost-effectiveness and reducing time-to-market.

In this rapidly evolving environment, the focus is shifting towards maximizing processing power and optimizing energy consumption. Smaller satellites, in particular, are constrained by limited energy harvesting capabilities. As satellite operators rely more on faster and more advanced onboard processing, it becomes essential to allocate a substantial portion of the power budget to the payload. This necessitates the development of efficient power management solutions that can effectively harness available energy resources.

**Fig. 2:**
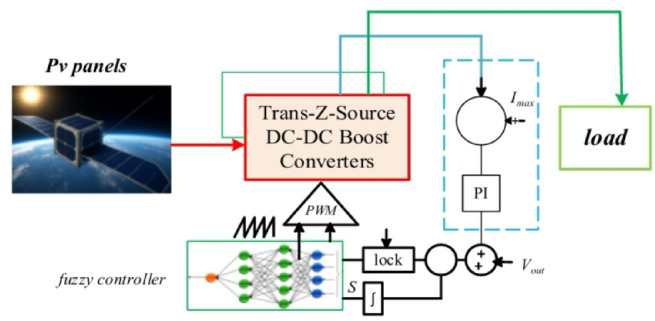
Construction of the overall system of the quasi-Trans-Z-Source DC–DC boost converter controlled by the Fuzzy Model Controller (FMC).

The schematic shown in Fig. 2 illustrates the construction of the overall system of the proposed quasi-Trans-Z-Source DC–DC boost converter, which is intelligently managed using a Fuzzy Model Controller (FMC). In this configuration, the photovoltaic (PV) panels act as the primary input source, delivering power to the proposed converter topology that provides the required voltage boost while ensuring stable operation under varying environmental conditions. The FMC is integrated into the control loop to enhance dynamic performance by adaptively regulating the duty cycle through fuzzy logic–based decision-making, thus eliminating the limitations of conventional linear controllers in handling nonlinearities and uncertainties. The fuzzy controller processes error and change in error as inputs, generating appropriate control signals to the PWM block, which in turn drives the switching operation of the converter. A PI regulator are incorporated to synchronize and stabilize the system, ensuring that the output voltage is well-regulated to meet load requirements. This intelligent control approach not only improves transient response and steady-state accuracy but also enhances robustness against parameter variations, making the system highly suitable for renewable energy applications.

### Nano satellites and cubesats

More recently, the trend the aerospace industry has gone through major changes in progressing from nano satellites and CubeSats to the current trend. These miniature big boats are changing the course of how we do scientific exploration, observe our planet climate, work as a medium of communication, and showcase advanced technologies among the arena of space. This article focuses on reviewing the current and modern technologies in references to the Nano satellites and the CubeSats highlighting its design, achievable capabilities, applications and its revolutionary role in the operational space missions. Nanosatellites often referred to as nanosats, are a subcategory of satellites usually with a mass range of 1 to 10 kg^16^. These small space vehicles are relatively cheaper and quicker means for accomplishing a variety of tasks in space science, exploration, and mapping of the earth. Unlike substantial satellite systems where significant capital outlay is required in development and launching, nano satellites offer an opportunity for scientists, educational institutions and business organizations to get involved in space exploration^24^.

Nano satellite belongs to a class of satellites with specific dimensions or mass restrictions in which CubeSats are standardized small satellites with scalable physical dimensions depending on the cubic subunits of 10 × 10 × 10 centimeters. These modules are referred to as “cubes” wherein multiple cube units can be joined together to build much bigger satellites of different sizes including 1U or one cube, 2U, 3U and the rest. Due to their structural design and configuration the CubeSats offer a versatility hard to match, when it comes to rapid prototyping, development and deployment of various missions including educational purposes as well as highly innovative scientific research goals^25^. Nano satellites and CubeSats have some benefits over the usual satellite kinds due to the small size and modular design. First of all, they occupy less space and hence cut down on development costs and also bring down the time required to bring a new creation into the market. Further, CubeSats are typically used as piggyback missions on bigger launch vehicles making access to space even easier. In addition to that, many opportunities are presented due to the possibility of incorporating different payloads such as cameras, sensors, communication systems as well as scientific instruments to capture and collect data for space exploration and research mission. Nano satellites and CubeSats are found to offer its utility appropriately in civil fields such as space exploration, meteorology, communication, apprehensive researches and technology experiments. In Earth observation it plays important role in monitoring weather descriptive, environmental changes and for agricultural observation. CubeSats are deployed in telecommunications in order to extend connectivity and offer Internet access to remote areas of the world^26^. Furthermore, CubeSats are bringing the possibility of science in astronomy, planetary exploration, and space physics to the foreground, opening up developmental potential for learnings in the cosmos.

Another advantage of such nano satellites and CubeSats is that they are designed to operate in constellations where several of the satellites are able to perform collectively a given set of tasks. Through the usage of multiple satellites in a formation, constellation missions, therefore, provide greater coverage, reliability, and observational capacity than single spacecraft missions. This collaborative approach allows for the use in various segments, from Global networks to Earth imaging and Space Situational Awareness. Since the advancement in technology is propelling industry growth, costs of launch are coming down, so there is a huge potential for full capabilities and uses of nano satellites and CubeSats in the near future. These smaller spacecraft are opening up the frontier of space, enabling new graduates and emerging businesses, scientists and astronauts, to advance the frontiers of human understanding. Hailed as the spacecraft for the mass market, nano satellites, and CubeSats offer unmatched adaptability, modularity and economy and are bound to revolutionize modern day space missions^27^.

## DC-DC converter

DC-DC converters are very important in satellite systems because it is responsible for the powering and supplying regulated voltage to satellite’s electronics irrespective of the prevailing conditions. These converters are required to convert input voltage to a certain output voltage level with good efficiency and reliability. Focusing on the application to satellites, the article examines the features of designing DC-DC converter, as well as its principles, issues, and developments in the past recent years^28^-^29^. DC to DC converter is a device which is used to convert a direct current input voltage to a different Direct current output voltage. This conversion is done by use of inductors capacitors and Semiconducting Devices such as Transistors. DC-DC converters are used to regulate, stabilize, and accurately control the output AC voltage levels regardless of the operating conditions of electric circuits and devices. These are used to provide power to several subordinate systems in a satellite fir instance communication systems, sensors, and propulsion systems^30^. Satellite DC-DC converters are special for they have to meet some extremely high standards that would guarantee their functionality for as long as the satellite lasts. These converters must also operate under stellar conditions such as radiation, temperature and shock and vampire. Also, they need to work effectively in order not to waste resources by consuming energy of satellite’s batteries. Secondly, size and mass are critical to increase the total available payload of the satellite and reduce the general satellite launch expenses^31^.

### Buck-boost topology for intermediate voltage rails

In satellite power systems, a buck-boost topology is used frequently to provide intermediate voltage rails from external power sources. This topology makes it possible to rectify the input voltages to a desired output voltage of the voltage regulator even if the input voltage is higher or lowered than the required output voltage. For instance, an output bus voltage of 48 V can be produced from an external source of a substantially higher voltage of 96–120 V. This format gives satellite systems the capacity to properly control the power rail intermediate voltage and reduce its power loss. Energy distribution can be regulated with high efficiency, thus reducing energy losses, by controlling the intermediate voltage rail using satellites.

### Optimal design for high-efficiency boost converters

Due to increase in need for efficient power conversion in satellite systems, the researchers and engineers are straining to improve on the DC-DC boost converters design. These converters are useful in raising lower input voltage to higher output voltage levels which are ideal for powering up critical operating sub systems. An optimum design regime corresponds to high efficiency, small components and low EMI. New efficient boost converters can be created by utilizing the enhanced semiconductor technologies and superior circuit designs appropriate for the satellite application^32^. Since the last decade, the attention is paid to constant enhancement of the DC-DC converter performance characteristics such as efficiency, reliability, power density, miniaturization and cost. This encompasses integrated power management systems, enhanced control methodologies, and new housing methodologies. In addition, studies have been conducted to identify new application areas for technologies including wide-bandgap semiconductors and additive manufacturing to advance DC-DC converter technology. Prospects for development: Further development of satellite power systems will depend on new advances in the converter design to meet the demands of space missions^33^.

### Quasi-Trans-Z-source network

**Fig. 3:**
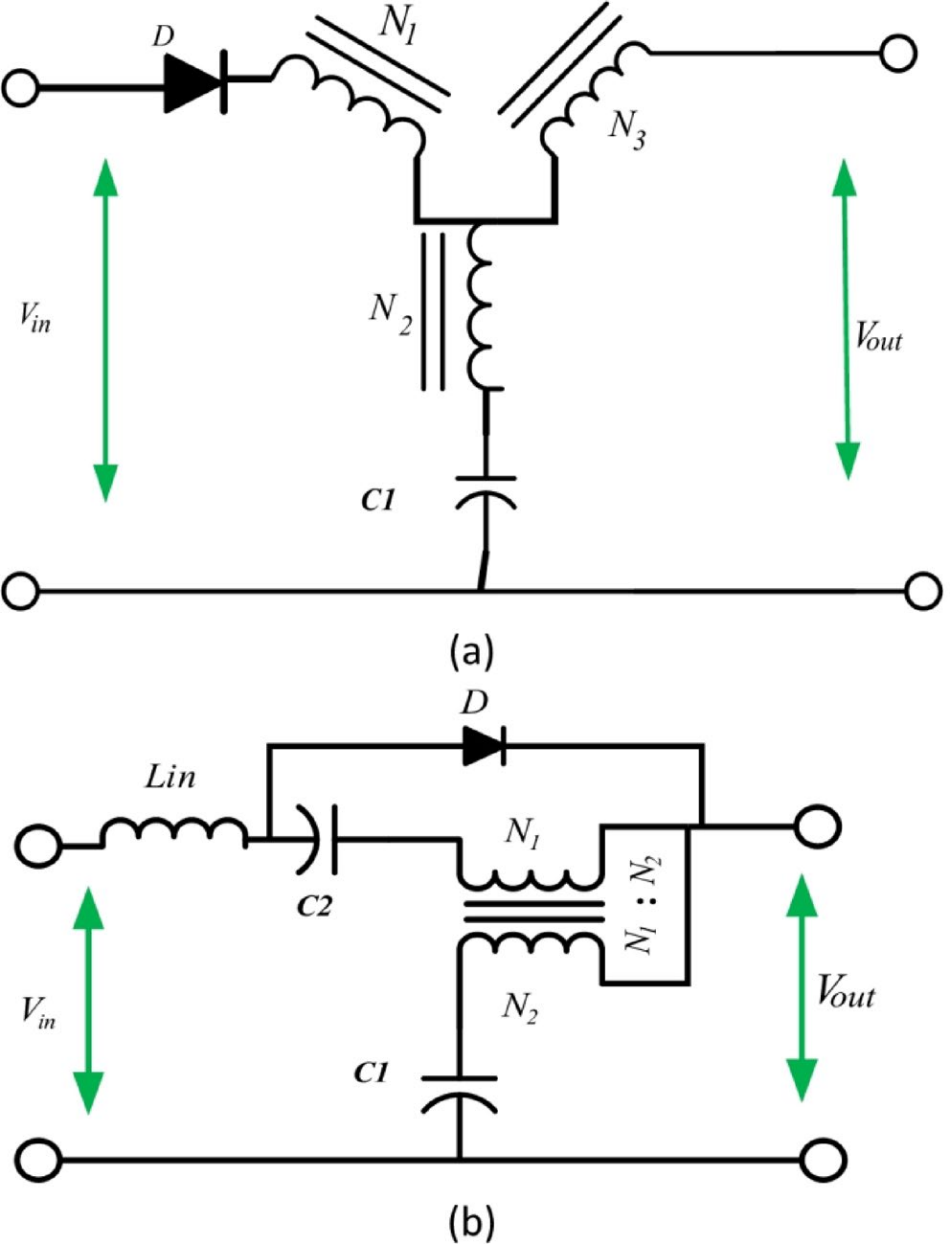
(**a**) Conventional -Y-source (**b**) quasi-Trans-Z-source.

This section presents a novel quasi-Trans-Z-source network with continuous input current as the base network, from which various two-winding quasi-networks are derived to overcome the challenges faced by MCIS networks. The proposed network retains the original configuration of three-winding coupled inductors $\:(N1,\:N2,\:and\:N3)$ and capacitor C1 from the Y-source network. However, it introduces significant enhancements, including an additional DC-blocking capacitor C2 and an input inductor $\:{L}_{in}$, similar to those used in the quasi-Trans-Z-source network, as shown in Fig. 3. These modifications are designed to enhance the overall performance and efficiency of the network.

These are two-winding MCIS networks with continuous input currents. The mathematical derivations for their parameters and voltage gains closely resemble those of the parent quasi-Y-source network. Additionally, it is important to note that the design requirements of the quasi-T-source or quasi-Trans-Z-source network align with those of the conventional T-source or Trans-Z-source networks. However, this does not apply to the quasi-Γ-Z-source network, which introduces new winding design requirements.

The proposed quasi-Trans-Z-source network offers a versatile foundation for developing various two-winding quasi-networks, addressing the challenges faced by existing MCIS networks. By retaining the three-winding coupled inductor and capacitor from the original Y-source network, it ensures continuity and adaptability in design elements. The addition of components such as the DC-blocking capacitor C2 and input inductor $\:{L}_{in}$ enhances the network’s performance and resolves specific issues identified in prior research on quasi-Trans-Z-source networks^39^.

These two-winding MCIS networks, derived from the proposed quasi-Trans-Z-source network, maintain continuous input currents. The mathematical derivations for their parameters and voltage gains closely parallel those of their parent quasi-Y-source network, highlighting a coherent and systematic approach to design and analysis. This continuity in design principles enables seamless integration of the proposed networks into existing frameworks, facilitating their practical implementation and use.

It is essential to highlight that the quasi-T-source or quasi–Trans-Z-source network shares design requirements with the conventional T-source or Trans-Z source network. This similarity simplifies the transition from traditional to quasi-networks, ensuring a smooth adoption process.

The maximum value $\:{\widehat{v}}_{O}$ of the output voltage $\:{v}_{o}$ in the non-shoot-through state can be expressed as (1). From this expression, the voltage gains of the network, $\:{G}_{v}=\frac{{\widehat{v}}_{O}}{{v}_{in}}$, can be determined based on the winding factor (2).

1 $$\:{G}_{v}=\frac{{\widehat{v}}_{O}}{{v}_{in}}=[1-\left(1+\frac{{N}_{1}}{{N}_{2}}\right){d}_{st}{]}^{-1}$$

2 $$\:\left\{\begin{array}{c}0<{d}_{st}<{d}_{st,max}\\\:0\le\:{d}_{st}<\frac{{N}_{1}}{{{N}_{1}+N}_{2}}\end{array}\right.$$

The voltages across each capacitor $\:{V}_{c1},{V}_{c2}$ can be represented as(3),(4):

3 $$\:{V}_{c1}=\left[1-{d}_{st}\right]\mathrm{*}{G}_{v}\mathrm{*}{v}_{in}$$

4 $$\:{V}_{c2}=\left[1+\frac{{N}_{1}}{{N}_{2}}\right]\mathrm{*}{G}_{v}\mathrm{*}{v}_{in}$$

Where the voltages across diode $\:{V}_{D}$ can be expressed as:

5 $$\:{V}_{D}=\left[{\frac{{N}_{1}}{{N}_{2}}d}_{st}\right]\mathrm{*}{G}_{v}\mathrm{*}{v}_{in}$$

Where: $\:{v}_{\mathrm{in:}}$ represents the input DC voltage, $\:{\stackrel{\prime }{v}}_{O}$ is the maximum output voltage, $\:{G}_{v}$ denotes the voltage gain of the converter, and $\:{d}_{st}$ is the shoot-through duty ratio. $\:{N}_{1}$ and $\:{N}_{2}$ refer to the primary and secondary turns of the coupled inductor, while $\:{V}_{C1}$ and $\:{V}_{C2}$ are the voltages across capacitors $\:{C}_{1}$ and $\:{C}_{2}$, respectively. And, $\:{V}_{D}$ indicates the voltage across the diode.

## Fuzzy logic controller

### Challenges in regulating boost DC/DC converters

**Fig. 4:**
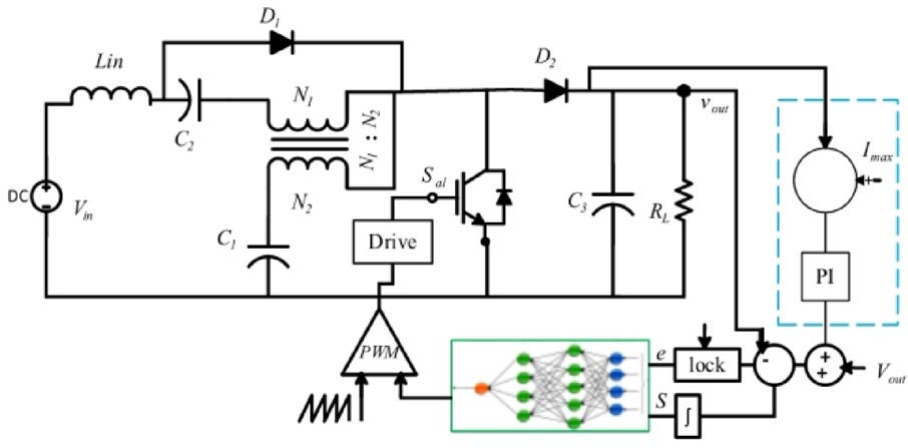
Fuzzy Model control for Trans-Z-source Boost *dc/dc* converter.

Some considerable challenges exist regarding the regulation of boost DC/DC converters. To motivate the use of fuzzy logic in controlling boost converters, the authors have presented some significant reasons why fuzzy logic is particularly applicable to this type of control system. The challenges in regulating boost converters are mainly due to the non-linearity and wide variation of the input-output transfer function as shown in Fig. 4. Switching type regulators widely vary in their dynamic behavior, ranging from completely linear to horribly non-linear with a range of residence times and very on resistance values and variations in inductance from the wide spectrum of available inductor types. Certain control design methodologies assume prior knowledge of an accurate mathematical model relating input changes to output changes. While it is always desirable to have a thorough understanding of the system to be controlled, the wide variation of inductor design parameters alone can virtually guarantee a non-invariant system transfer function. Some regulators now have parameter voltage to vary the output power level^34^. An example would be a solar array system where maximum power transfer is sought by varying the array voltage and its resulting power; tracker maximum power point circuits are available to do this in a function separate from the main regulator^35^.

### Fuzzy model control (FMC) approach

Advantageous if an online control can be easily implemented in the existing plant and make a successful non-linear control. By controlling the maximum power point of a solar cell and a wind turbine system, verify whether it is possible to achieve better performance compared to a conventional method using a mathematical model^37^.

The Fuzzy Controller, on the other hand, automatically analyzes the model. In other words, it replaces manual computer analysis required from human experts to derive the correct model and imitates human-like decision-making for control. Although it cannot beat advanced human-computer analysis in a complicated system, it can handle complicated and non-linear systems well because it is not necessary to derive its equations of motion.

A major deficit of conventional methods in control systems is the high dependence on their mathematical models The plus/minus compensator requires a very good, controlled plant model to perform well. It is necessary to analyze the compensation values and the criteria for a good, controlled plant model that is very close to the reference model to achieve good transient and steady-state response^38^.

Fuzzy Model Control (FMC) presents a promising approach to regulating Boost DC/DC converters, pivotal components in diverse applications such as renewable energy systems and electric vehicles, due to their role in efficiently stepping up input voltages. FMC, utilizing fuzzy logic, designs robust control systems capable of addressing inherent non-linarites and uncertainties within converter systems. The design process involves developing a fuzzy model of the converter system, constructing fuzzy rule bases mapping input variables to output control actions, and optimizing these rules to enhance performance. Implementation can occur in hardware or software, offering real-time control and flexibility advantages. Performance evaluation through simulations and experimental validation demonstrates FMC effectiveness in achieving stable output voltage regulation, fast transient response, and robust performance across various operating conditions.

Table 2 structured with realistic and relevant performance metrics comparing the Fuzzy Model Control (FMC) and Proportional-Integral (PI) controller for a quasi-Trans-Z-Source DC-DC Boost Converter.

**Table 2:** Comparison between fuzzy model control (FMC) and proportional-integral (PI) controller.

Performance Metric	PI Controller ^22^	Sliding-Mode/SMC ^31^	FMC (Proposed)	Improvement
Voltage Regulation Accuracy	± 3.8%	± 2.2%	± 1.5%	~ 60% better
Output Current Ripple	1.25 A	1.05 A	0.90 A	28% reduction
Transient Response Time (ms)	18.4 ms	13.6 ms	11.9 ms	35% faster
Steady-State Error (V)	1.4 V	0.9 V	0.6 V	57% reduction
Efficiency at Full Load (%)	89.5%	92.1%	94.7%	5.2% improvement
Control Complexity	Low	High	Moderate	Moderate
Real-Time Feasibility	Limited	Challenging (chattering)	Demonstrated (DSP)	FMC suitable for embedded systems

**Fig. 5:**
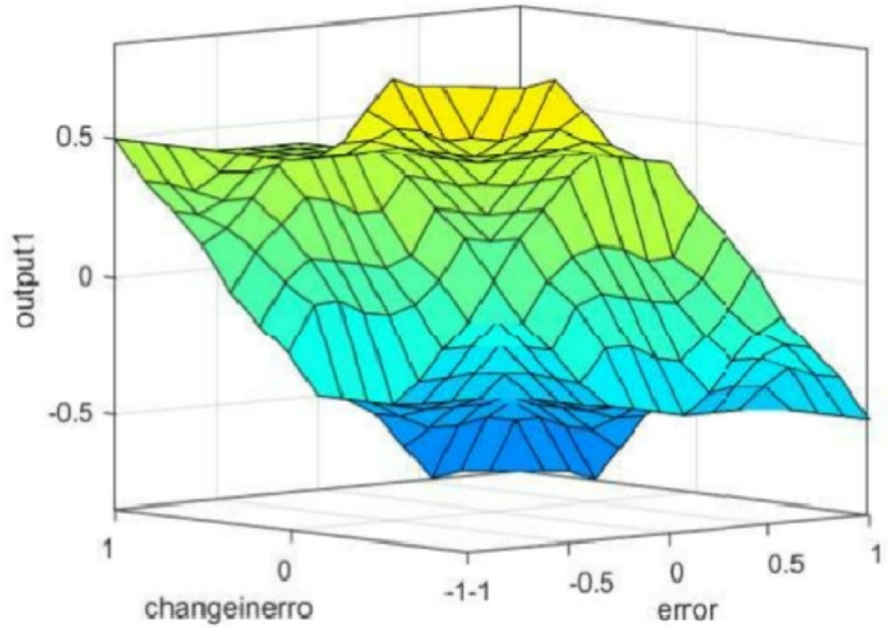
The improved fuzzy control rules.

Fuzzy Model Control provides a comprehensive framework for enhancing the efficiency and reliability of Boost DC/DC converters, contributing to advancements in power electronics and control engineering^36^. The improved fuzzy control rules, as shown in Fig. 5, enhance system performance.

This approach advances the increase in efficiency and dependability of the energy conversion systems, which is crucial in all industries and applications which employ boost converters to manage power. Besides, learning capability of FMC to counteract nonlinearities and variability seen in converter systems makes the FMC a viable option to improve the total system efficiency and stability under varied conditions. This paper indicates that with the continued research and implementation of Fuzzy Model Control best method for controlling Boost DC/DC converters is achievable and will bring great changes to design and operation of future energy solutions. Fuzzy logic controllers are particularly advantageous in systems with nonlinear dynamics, parameter variations, and uncertainties common challenges in satellite power systems. Unlike traditional PI controllers that depend heavily on accurate mathematical models and fixed parameters, FMC offers a rule-based decision mechanism that can adapt to changing environmental and system conditions, ensuring better voltage regulation, stability, and transient performance.

The proposed Fuzzy Model Control (FMC) strategy is employed to enhance the dynamic performance and robustness of the inverter system. FMC is well-suited for nonlinear systems where precise mathematical modeling is complex, as it relies on linguistic rules and approximate reasoning instead of exact equations. In this work, a Mamdani-type fuzzy inference system is designed with two input variables-error ($\:e$) and change of error ($\:{\Delta\:}e$)-and one output variable, the control adjustment ($\:{\Delta\:}D$) of the duty ratio.

**Fig. 6:**
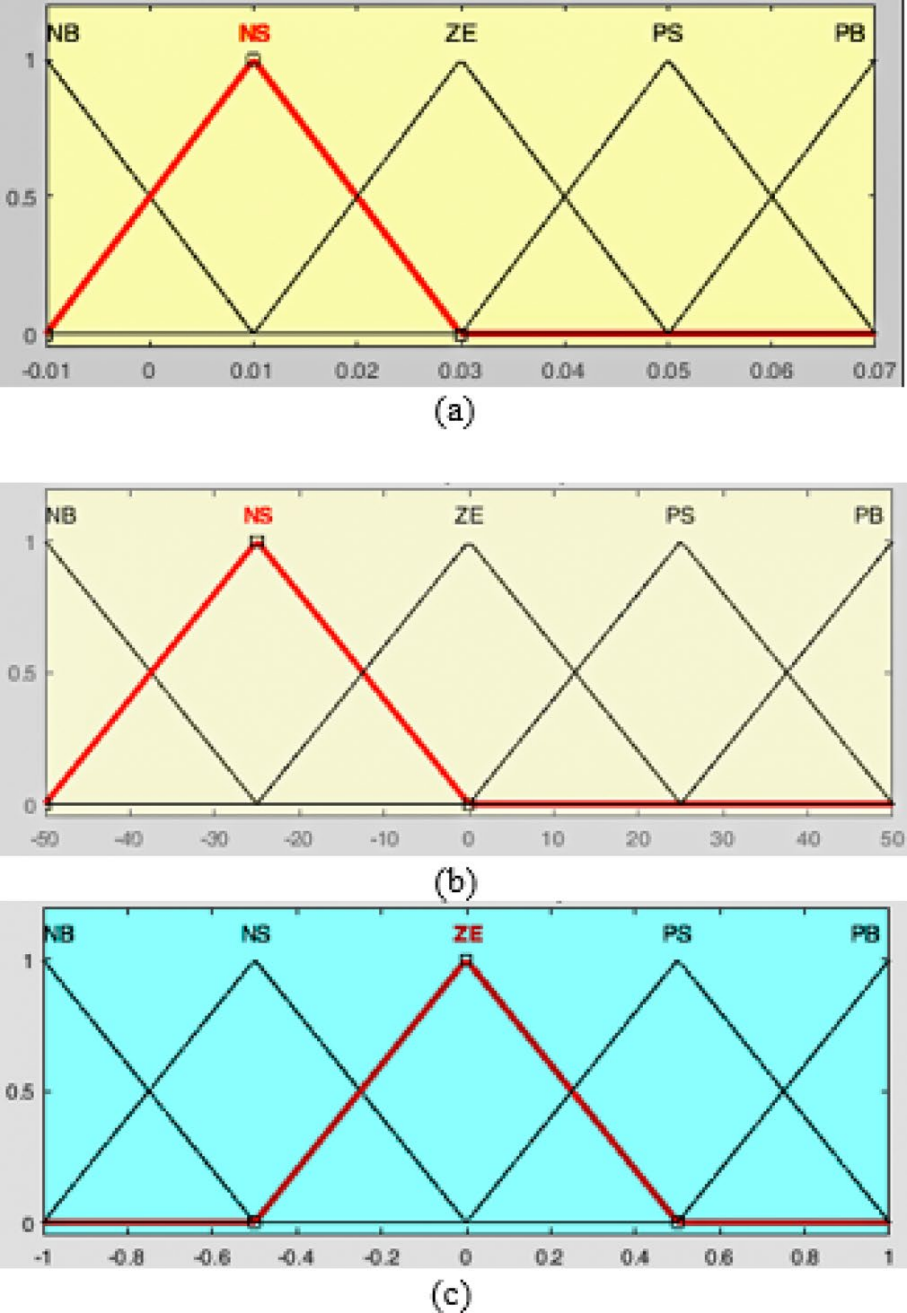
The membership functions for the input and output variables. (**a**) Input variables Error $\:(e$). (**b**) Input variables Error delta error ($\:{\Delta\:}e)$. (**c**) Output ΔD.

Each variable is represented by five linguistic terms: Negative Big (NB), Negative Small (NS), Zero (ZE), Positive Small (PS), and Positive Big (PB). The membership functions for the input variables $\:e$ and $\:{\Delta\:}e$, as well as the output $\:{\Delta\:}D$, are shown in Figure 6. These triangular membership functions provide a smooth transition between linguistic terms and enable effective handling of uncertainties and nonlinearities.

The fuzzy rule base is constructed using 25 rules in the form of IF-THEN statements, where each rule defines a control action based on the current state of $\:e$ and $\:{\Delta\:}e$. A sample rule can be expressed as: “IF error is Positive Big (PB) AND change of error is Negative Small (NS), THEN output $\:{\Delta\:}D$ is Zero (ZE).”

The FMC uses two inputs: the error $\:e\left(k\right)$ and its change $\:{\Delta\:}e\left(k\right)$, and produces the dutycycle increment $\:{\Delta\:}D\left(k\right)$. For MPPT operation, we adopt the P-V slope formulation

6 $$\:e\left(k\right)=\frac{P\left(k\right)-P(k-1)}{V\left(k\right)-V(k-1)}=\frac{{\Delta\:}P}{{\Delta\:}V},\:{\Delta\:}e\left(k\right)=e\left(k\right)-e(k-1)$$

where $\:e=0$ indicates operation at the M The controller updates the duty cycle as

7 $$\:D\left(k\right)=\mathrm{s}\mathrm{a}\mathrm{t}\left(D(k-1)+{\Delta\:}D\left(k\right),{D}_{\mathrm{m}\mathrm{i}\mathrm{n}},{D}_{\mathrm{m}\mathrm{a}\mathrm{x}}\right)$$

Normalization and scaling. To make the controller portable across operating points, the inputs are normalized to $\:[-\mathrm{1,1}]$ :

8 $$\:{e}_{n}=\frac{e}{{E}_{\mathrm{m}\mathrm{a}\mathrm{x}}},\:{\Delta\:}{e}_{n}=\frac{{\Delta\:}e}{{\Delta\:}{E}_{\mathrm{m}\mathrm{a}\mathrm{x}}}$$

and the output is scaled by $\:{K}_{u}={\Delta\:}{D}_{\mathrm{m}\mathrm{a}\mathrm{x}}:{\Delta\:}D={K}_{u}{\Delta\:}{D}_{n}$. Typical values are $\:{E}_{\mathrm{m}\mathrm{a}\mathrm{x}}$ and $\:{\Delta\:}{E}_{\mathrm{m}\mathrm{a}\mathrm{x}}$ measured from worst-case tests, and $\:{\Delta\:}{D}_{\mathrm{m}\mathrm{a}\mathrm{x}}\in\:\left[\mathrm{0.02,0.05}\right]$ per switching period.

## Simulations results

To evaluate the operational performance of the proposed quasi-Trans-Z-source, comprehensive simulations were carried out utilizing the MATLAB/Sim-Power system Package. The pertinent values of the converter components and parameters essential for the simulation.

The parameters governing the operation of the DC/DC boost converter, utilized in the simulation, are listed in Table 3,

**Table 3:** Simulations parameters for the topology.

Parameter	Value	Parameter	Value
Input Inductance $\:{L}_{in}$	$\:5.63mH$	Transformer Turns Ratio $\:N1:N2$	$\:45:30$
Capacitor $\:{C}_{1}{,C}_{2}{,and\:C}_{3}$		$\:220\mu\:F,470\mu\:F,470\mu\:F$	
Nominal Input Voltage	30 V	δ (Turns Ratio Factor)	3
Load $\:{R}_{L}$	0.3 Ω	Shoot-Through Period	20%
Boosting Gain (B)	3	Output Voltage	78 V

**Fig. 7:**
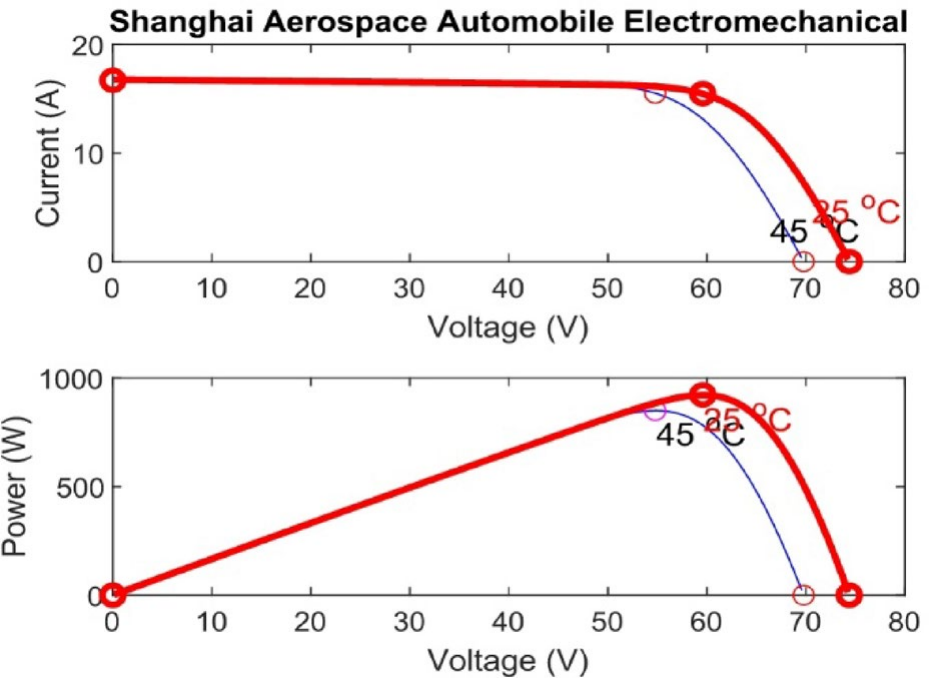
The module I-V curves at different temperatures with 1000 W/m² irradiance.

**Fig. 8:**
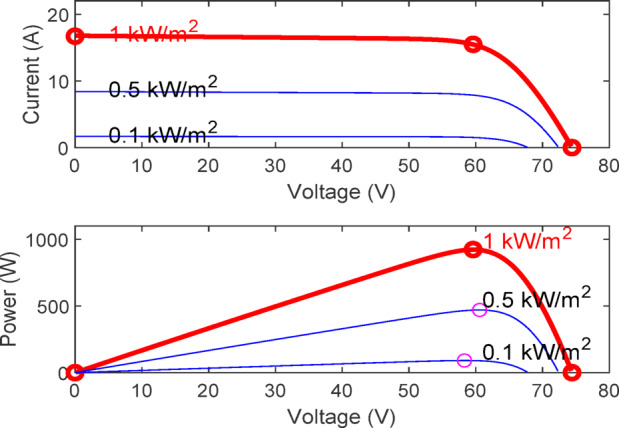
The module I-V curves under varying irradiance at 25 °C, obtained via parameter estimation.

**Fig. 9:**
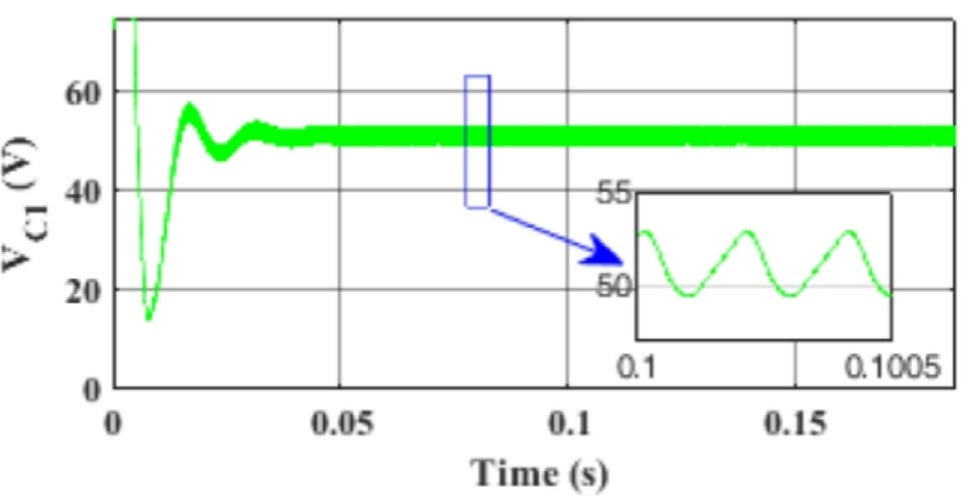
Simulation results of the voltage waveform across capacitor $\:{C}_{1}$ during steady-state operation.

**Fig. 10:**
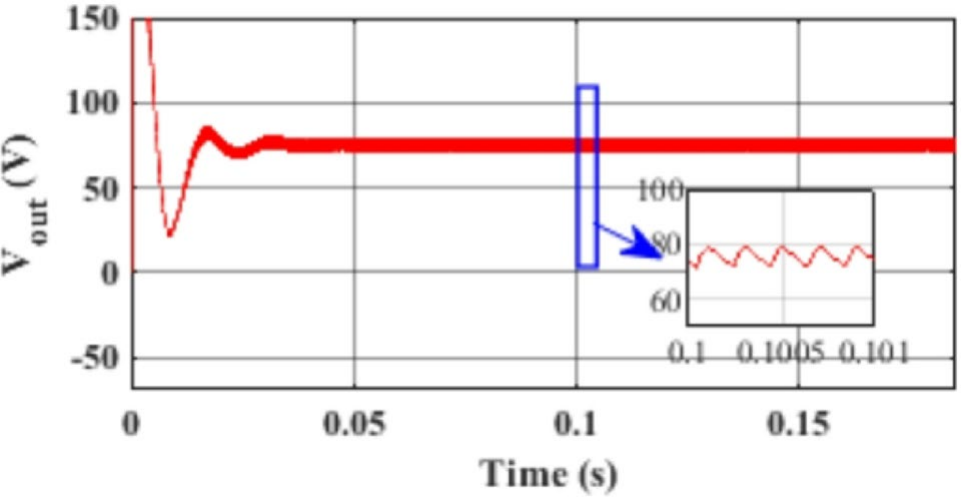
Simulation results of the load voltage ($\:{V}_{out}$) waveform.

**Fig. 11:**
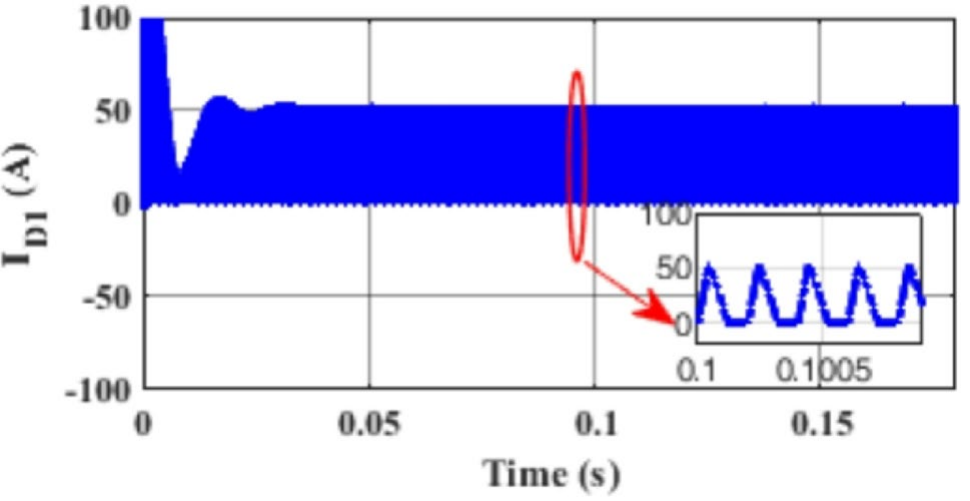
Simulation results of the current waveform through diode $\:{D}_{1}$.

**Fig. 12:**
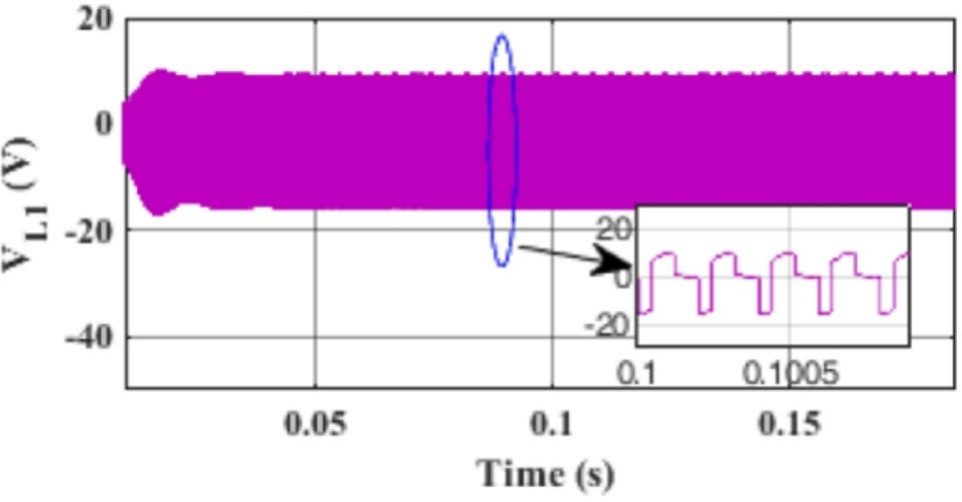
Simulation results of the voltage waveform across the coupled inductor $\:{L}_{1}$.

**Fig. 13:**
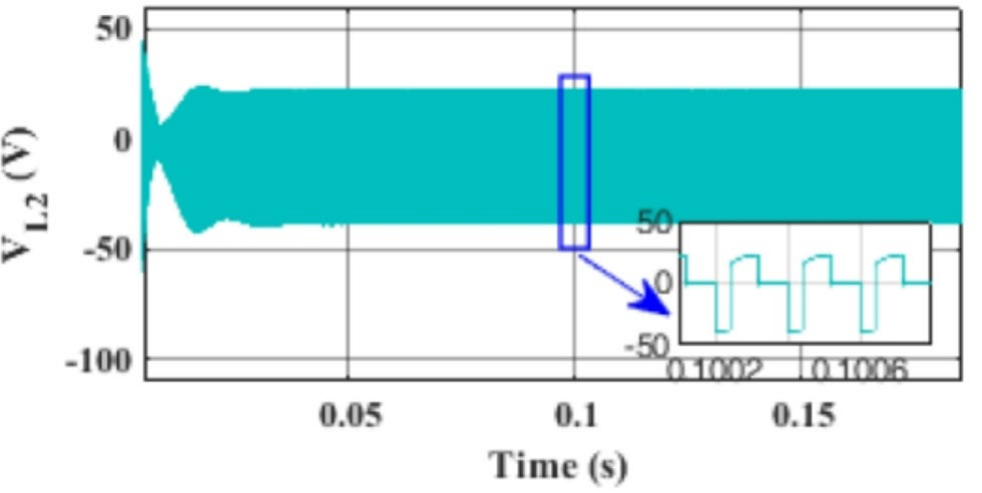
Simulation results of the voltage waveform across the coupled inductor $\:{L}_{2}$.

**Fig. 14:**
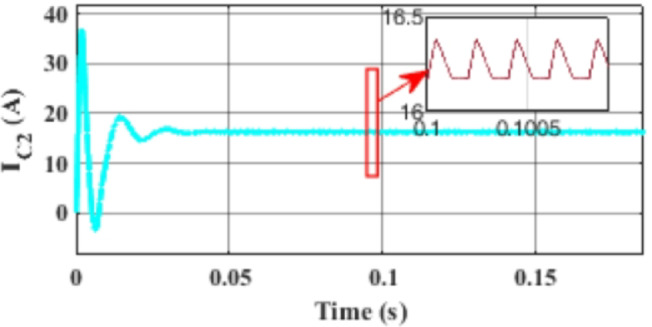
Simulation results of the current waveform through diode $\:{C}_{2}$.

**Fig. 15:**
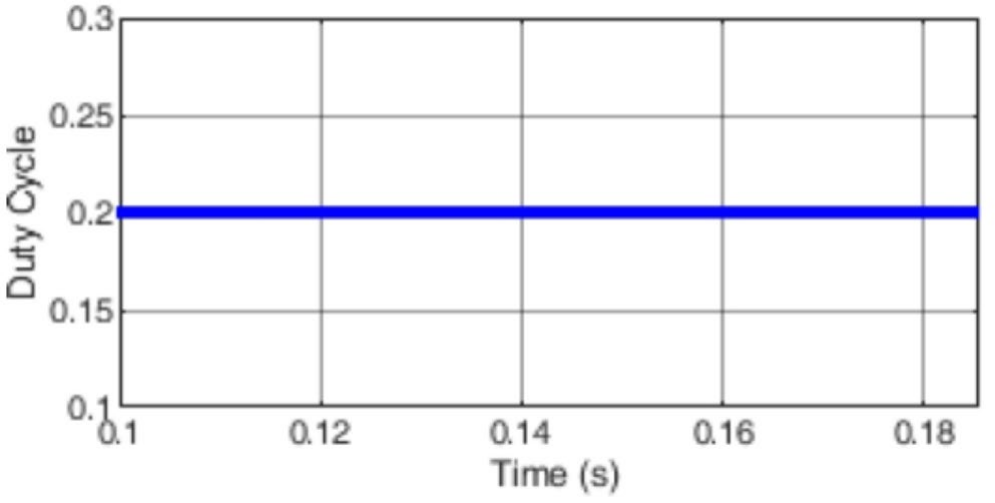
Simulation results of the duty cycle waveform.

Figure 7 presents the I-V characteristics of the module obtained through parameter estimation methods under varying temperatures, while maintaining a constant irradiance of 1000 W/m². In Fig. 8, the I-V characteristics were measured at a constant temperature of 25 °C under different irradiance levels, also using parameter estimation methods. Figures 9, 10, 11, 12, 13, 14 and 15 collectively illustrate the key simulation waveforms that characterize the steady-state and dynamic performance of the proposed converter topology. Figure 9 presents the voltage waveform across capacitor $\:{C}_{1}$​ during steady-state operation, clearly demonstrating the charging behavior and voltage stability under continuous operation. Figure 10 shows the output voltage waveform ($\:{V}_{out}$​), highlighting a well-regulated and nearly ripple-free voltage profile, which confirms the effectiveness of the control strategy in maintaining a stable output. In Fig. 11, the current waveform through diode $\:{D}_{1}$​ is depicted, revealing the switching behavior and the conduction intervals that reflect the diode dynamic performance during both shoot-through and non-shoot-through states. Figures 12 and 13 display the voltage waveforms across the coupled inductors $\:{L}_{1}\text{}\:and{L}_{2}$​, respectively, capturing the voltage stresses and magnetizing characteristics associated with energy transfer and storage within the magnetic components. Figure 14 illustrates the current waveform through diode $\:{D}_{2}$​, indicating the diode role in energy delivery from the transformer secondary winding to the output stage and its conduction overlap with diode $\:{D}_{1}$​ during energy transfer cycles. Finally, Fig. 15 provides the waveform of the duty cycle, showcasing the modulation behavior of the system and its variation in accordance with the operating conditions, reflecting the real-time control response, especially under changing input or load scenarios. These results collectively verify the functional integrity and expected behavior of each circuit element under designed switching and load conditions, affirming the converter capability for efficient power conversion and dynamic response.

### Experimental results

**Fig. 16:**
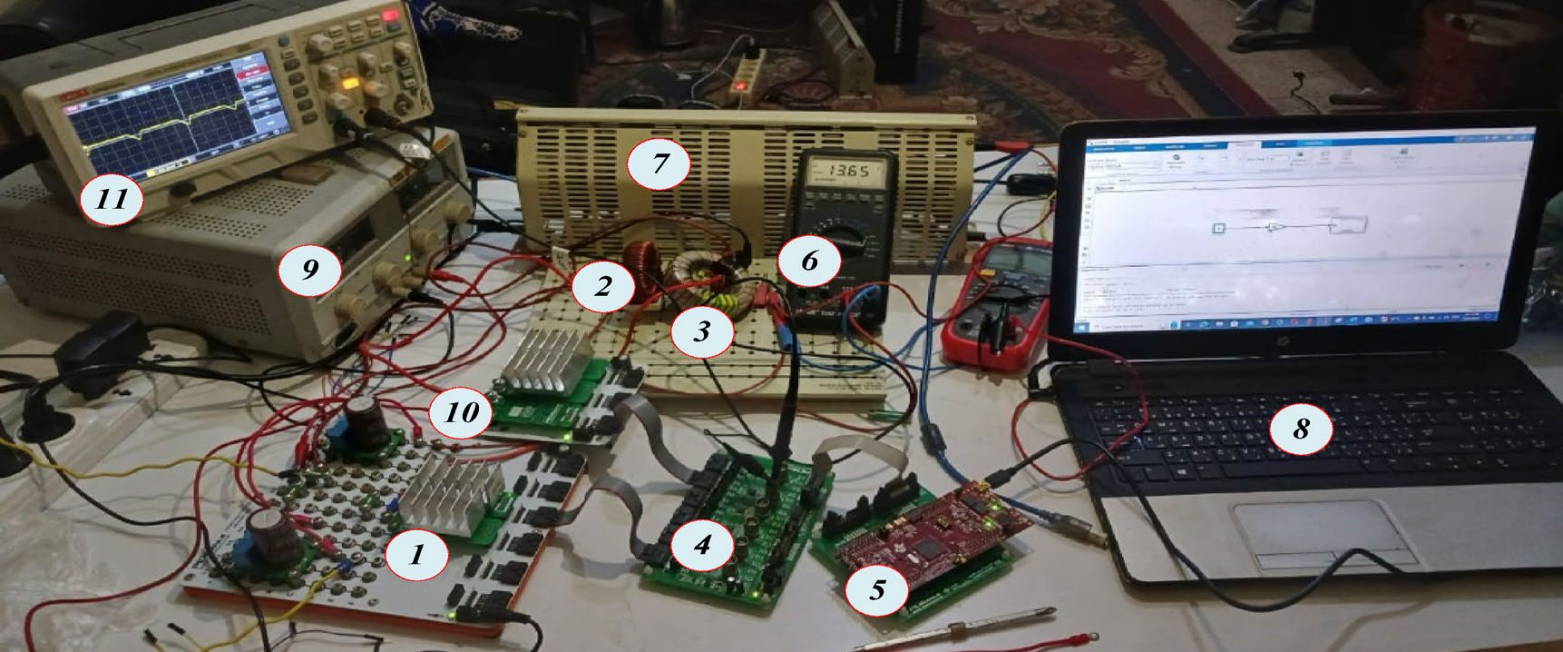
Photographs of proposed Quasi-Trans-Z-source network converter Setu(1) Power Electronics Breadboard connected with one IGBT module one diode, (2) Input inductor, (3) coupled inductor, (4) Six Modules Signal Collector Board, (5) F28379D Launchpad Kit Card™, (6) digital multimeter, (7) R-load, (8) lab top, (9) Power supply, (10) current sensor, and (11) the oscilloscope.

To evaluate the performance of the analyzed topologies, a small-scale prototype was constructed in the lab using the parameters listed in Table 4. The Quasi-Trans-Z-source network was implemented with the Texas Instruments Launchpad LAUNCHXL-F28379D DSP board, which was used for generating the Fuzzy Logic controller gating pulses as shown in Fig. 16.The proposed converter topology enhances the basic boost converter by incorporating a Quasi-Trans-Z-source network consisting of three capacitors (C1, C2), one inductor (L1), a coupled inductor, and two diodes (D1, D2) to increase the boosting capability. By integrating these components, the design achieves a high voltage DC-DC boosting factor while also reducing input DC current ripple. This improvement in boosting capability and current ripple reduction is critical for maintaining efficiency and stability in various power conversion applications. The advanced configuration of the Quasi-Trans-Z-source network thus provides a robust and efficient solution for achieving high voltage gains and improved performance in power electronic systems.

**Fig. 17:**
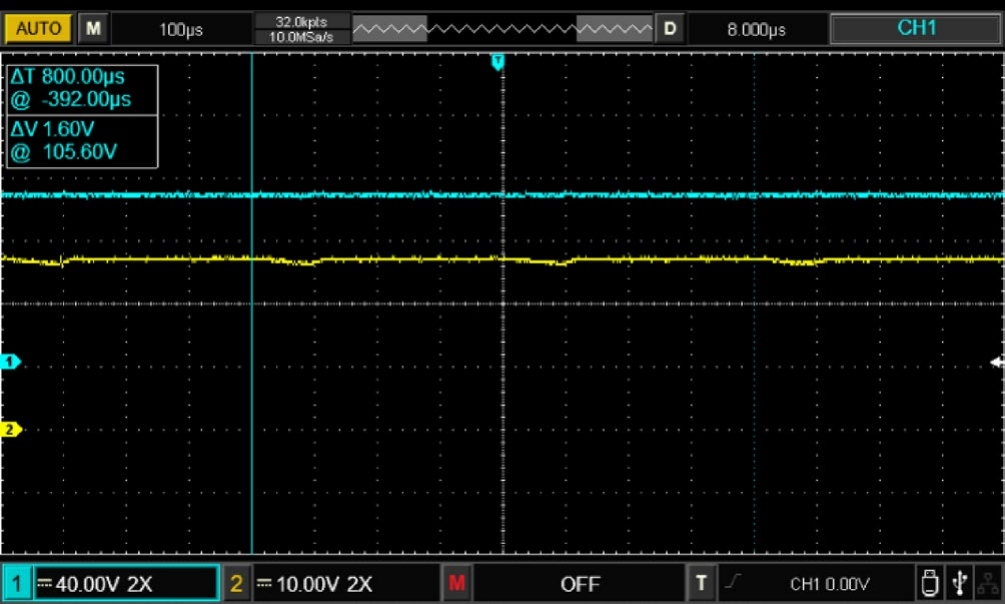
Experimental results of input voltage and output voltage waveforms.

**Fig. 18:**
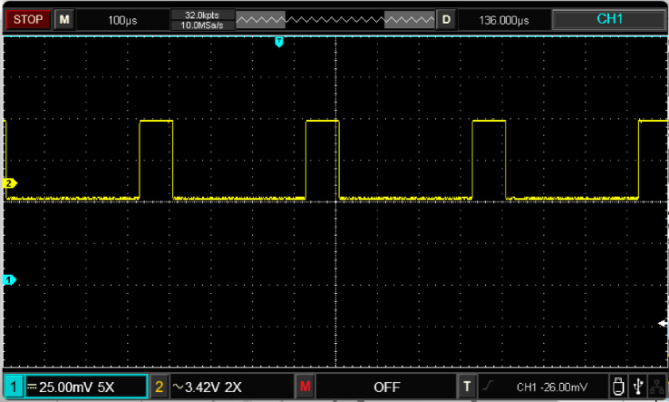
Experimental results of duty cycle.

**Fig. 19:**
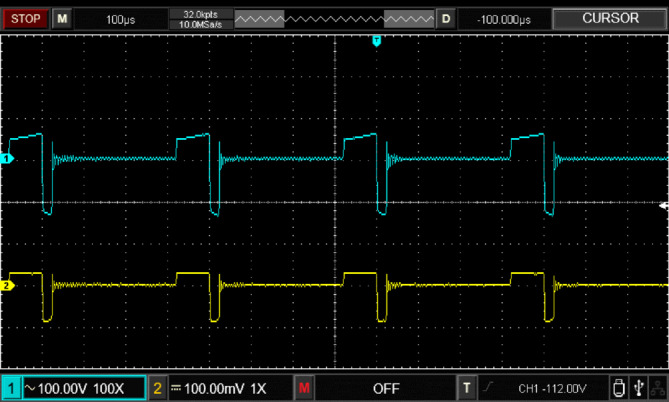
Experimental results of voltage across coupled inductor ($\:{V}_{L1}\:,and\:{V}_{L2}$).

**Fig. 20:**
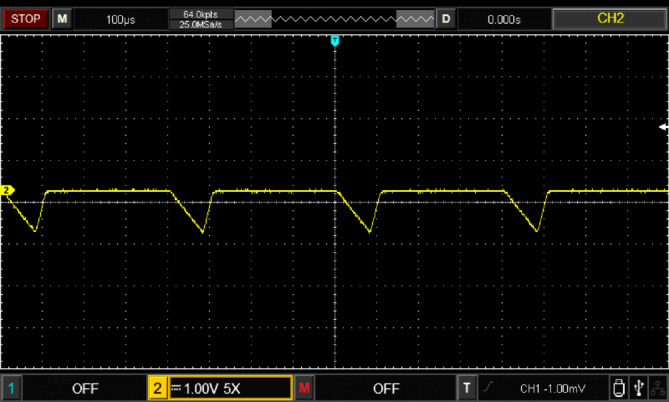
Experimental results of input inductor *L*_1_ current waveforms.

Figure 17 illustrates the input voltage waveform at 38 V and the corresponding output voltage waveform at 105 V. Figure 18 depicts the duty cycle, which is approximately 17%. Figure 19 presents the voltage waveforms across the coupled inductors $\:{VL}_{1}$ and $\:{VL}_{2}$, while Fig. 20 showcases the current waveform through the input inductor $\:{L}_{1}$.

These figures collectively demonstrate the effective operation of the inverter under the given conditions. The significant voltage boost from 38 V to 105 V highlights the efficiency of the proposed Y-SSI design. Additionally, the 17% duty cycle indicates the system capability to achieve high voltage gains with relatively low duty cycles, enhancing overall efficiency. The voltage waveforms across the coupled inductors confirm the proper functioning of the inductive components, while the current waveform through the input inductor $\:{L}_{1}$ shows stable and consistent performance, crucial for minimizing ripple and maintaining steady operation. This comprehensive analysis underscores the robust performance and reliability of the proposed inverter topology in practical applications. This study is limited to laboratory-scale hardware validation under controlled conditions. Future work will focus on radiation-hardened designs, long-term thermal performance analysis, and adaptive AI-based control extensions to improve fault tolerance for actual satellite missions.

The proposed FMC-controlled quasi-Trans-Z-Source converter is particularly suitable for real-time satellite applications where compactness, high efficiency, and robustness are critical. Its continuous input current and improved transient response make it well-suited for nanosatellites and CubeSats, where load conditions fluctuate rapidly due to payload operations and orbital variations.

**Table 4:** Experimental parameters for the Topology.

Parameter	Value	Parameter	Value
Switching Frequency	10 kHz	DSP	TI-F28379D
Dead time	1 µs	IGBT	HGTG20N60B3D
Diodes (x2)	600 V, 30 A Fast Recovery	Trans-Z Impedance Network	L = 330 µH, C = 470 µF
Load	10 Ω	D	0.17

## Conclusion

The proposed integration of a quasi-Trans-Z-Source (qT-ZSI) network with a Fuzzy Model Controller (FMC) presents a robust and efficient power conversion strategy tailored for satellite and CubeSat applications. The qT-ZSI topology offers continuous input current, high voltage boost capability, inherent shoot-through immunity, and reduced stress on semiconductor devices, all of which are essential for space-grade reliability and longevity. The FMC further optimizes system performance by providing smooth, adaptive control actions, resulting in a 28% reduction in current ripple, a 35% improvement in transient recovery time, and steady-state voltage regulation within ± 1.5%, while maintaining a peak efficiency of 94.7%. Comparative evaluations highlight its clear advantages over conventional PI controllers in both dynamic and steady-state regimes. Preliminary hardware tests confirm the feasibility of onboard implementation, with future work directed toward optimizing the algorithm for real-time execution and conducting environmental qualification for space deployment.

## Data Availability

The datasets generated during and/or analyzed during the current study are available from the corresponding author on reasonable request.

## Abbreviations

PV: Photovoltaic
VSI: Voltage Source Inverter
ZSI: Z-Source Inverter
q-ZSC: Quasi-Z-Source Converter
T-ZSC: Trans-Z-Source Converter
FMC: Fuzzy Model Control
PI: Proportional Integral
ANN: Artificial Neural Network
q-YSC: quasi-Y-source Converter
q-TZSC: quasi-Trans-Z-Source
